# Low Back Pain in Adolescents: Associations With Demographics, Physical and Psychosocial Factors, and Magnetic Resonance Imaging Features of the Spine

**DOI:** 10.1097/BRS.0000000000004690

**Published:** 2023-05-11

**Authors:** Marleen M. van den Heuvel, Alessandro Chiarotto, Edwin H.G. Oei, Marienke van Middelkoop

**Affiliations:** a Department of General Practice, Erasmus MC University Medical Center Rotterdam, Rotterdam, The Netherlands; b The Generation R Study Group, Erasmus MC University Medical Center Rotterdam, Rotterdam, The Netherlands; c Department of Radiology and Nuclear Medicine, Erasmus MC University Medical Center Rotterdam, Rotterdam, The Netherlands

**Keywords:** adolescents, imaging, pain, low back

Low back pain (LBP) is reported as a common complaint in adolescents and can negatively impact their physical and mental health. A wide range of biological and social factors may play a role in the development of adolescent spinal pain. Mental well-being and overweight are factors in the literature consistently associated with LBP.^1^

The pathophysiological mechanisms of LBP have been investigated in a limited manner in young populations. We recently showed that, in an open population-based cohort study, structural spinal abnormalities on magnetic resonance imaging (MRI), especially disc bulging, endplate irregularities, and abnormal disc height, are already present in children aged 9 years.^2^ Of these abnormalities, endplate irregularities are associated with various weight and body composition measurements. To better understand the future consequences of these abnormalities, the present study aims to investigate the prevalence and characteristics of LBP at the age of 13 years. Moreover, we aimed to assess associations of demographics, physical and psychosocial factors, and MRI features of the spine (*i.e.* structural abnormalities, vertebral shape, and spinopelvic alignment) at the age of 9 years with LBP at age 13.

## MATERIALS AND METHODS

Data from the Generation *R* Study, a population-based birth cohort, were used for this study.^3^ In Generation *R*, 9749 children were included and regularly followed from fetal life onwards. All children participating in the study at the age of 13 years were invited for an MRI scan, a short interview on the presence and characteristics of pain, and anthropometric measurements (to calculate body mass index SD-score). To assess the presence of pain, children were asked whether they had pain in the past 6 weeks for more than half of the days. If so, the location was checked on a pain mannequin, from which LBP data were extracted. Data on demographics (sex and maternal educational level), physical activity (sports participation), sedentary behavior (television viewing/computer game use), psychosocial factors (the youth self-report questionnaire on behavioral problems), and presence of musculoskeletal (MSK) pain at the age of 6 years were available from questionnaires. The MRI measurements included spinal abnormalities, vertebral shape, and spinopelvic alignment at the age of 9 years.^2^,^4^ These MRI measurements were available for a selection of the participating children (n = 409) based on the availability of accelerometer data, which did not include all children with LBP. Univariable logistic regression was used to assess associations of physical factors, psychosocial factors, MRI features at 9 years, and MSK pain at 6 years, with LBP at the age of 13 years. Analyses were performed using IBM SPSS Statistics for Windows, Version 28.0 (Armonk, NY: IBMCorp).

## RESULTS

Out of the 9749 children participating in the Generation *R* Study, data on pain at the age of 13 years were available for 3062 children. Of them, 714 (23.3%) of the children reported any pain, of whom 575 children reported MSK pain (18.8%) and 69 children (2.3%) reported LBP. Therefore, 80.5% and 9.7% of the children with pain had MSK pain or LBP, respectively. Over half of the children with LBP experienced pain daily (54.3%) and 92% reported a pain duration of more than 3 months. The pain often had a sudden onset (62.9%) and was related to sports in 42.9% of the cases. The mean LBP intensity was 5.9 (SD 1.5) on a 0 to 10 scale.

Boys experienced less frequent LBP, whereas no associations were found for the maternal educational level, body mass index SD-score, physical activity, and sedentary behaviors (Table 1). Children with LBP had a higher score on behavioral problems compared with children without LBP. A larger number of children with LBP (53.6%) reported pain at more than one location of the body. No associations were found between MSK pain or LBP at the age of 6 years and LBP at the age of 13 (Table 1).

**TABLE 1:** Characteristics of Participants and Association With the Presence of LBP (N = 3062)

	LBP at Age 13 y		OR for LBP (95% CI)
	Yes (n = 69)	No (n = 2993)	
Demographics			
Sex (M)	20 (29.0)	1448 (48.4)	**0.44** (**0.26–0.74)** *
Educational level mother			
** **Low	19 (34.5)	1009 (38.8)	Reference
** **Intermediate	16 (29.1)	762 (29.3)	1.12 (0.57–2.18)
** **High	20 (36.4)	832 (32.0)	1.28 (0.68–2.41)
Weight status			
** **BMI, SD-score	0.53 (1.21)	0.43 (1.19)	1.07 (0.88–1.30)
Physical activity/sedentary behavior			
Sports participation (yes)	46 (82.1)	2114 (84.9)	0.82 (0.41–1.64)
Television viewing (h/d)	1.50 (0.50–1.79)	0.79 (0.50–1.79)	1.26 (0.97–1.64)
Computer game use (h/d)	3.00 (1.79–4.07)	3.00 (2.00–4.43)	0.92 (0.78–1.08)
Psychosocial factors			
YSR total problems sum score (0–210)	34.0 (22.5–49.0)	29.0 (18.0–41.4)	**1.01** (**1.00–1.03)** *
Multiple pain locations			
>1 pain location (yes)	37 (53.6)	283 (9.5)	**11.07** (**6.79–18.05)** †
History of pain			
MSK pain at age 6 y (yes)	7 (12.3)	269 (10.4)	1.20 (0.54–2.68)
LBP at age 6 y (yes)	0	18 (0.6)	NA

Values are presented as number (%) for categorical factors and median (interquartile range) or mean (SD) for continuous factors. This table is based on nonimputed data; missing data were 0 for sex, 404 (13.2%) for educational level mother, 2 (0.1%) for body mass index, 516 (16.9%) for sports participation, 859 (28.1%) for television viewing, 898 (29.3%) for computer game use, 457 (14.9%) for youth self-report total problem score, 0 for multiple pain locations, 426 (13.9%) for musculoskeletal pain at age 6 years, and 426 (13.9%) for low back pain at age 6 years.

Bold values represent statistically significant odds ratios.

* *P* < 0.05.

† *P* < 0.001.

BMI indicates body mass index; LBP, low back pain; MSK, musculoskeletal; NA, not available; OR, odds ratio; YSR, youth self-report.

Table 2 shows that none of the structural spinal abnormalities on MRI at the age of 9 years was associated with LBP at the age of 13 years. This also applies to the vertebral shape, where no associations were found for the different tertiles of the vertebral concavity ratios at none of the levels, as illustrated by level L5 in Table 2. Lastly, we did not find an association between the pelvic incidence tertiles and the presence of LBP.

**TABLE 2:** Association Between MRI Features at Age 9 Years and the Presence of LPB at Age 13 Years (n = 409)

	LBP at Age 13 y		OR (95% CI)
	Yes (n = 13)	No (n = 396)	
Structural spinal abnormalities			
Signal intensity, abnormal	2 (16.7)	100 (26.0)	0.47 (0.12–2.64)
Disc height, decreased	4 (33.3)	153 (39.7)	0.76 (0.22–2.56)
Pfirrmann grade, abnormal	1 (8.3)	14 (3.7)	2.37 (0.29–19.66)
Nuclear shape, abnormal	4 (33.3)	120 (31.3)	1.10 (0.33–3.72)
Disc bulging, present	9 (75.0)	283 (74.7)	1.02 (0.27–3.84)
Endplate irregularities, present	5 (38.5)	151 (40.2)	0.93 (0.30–2.90)
Vertebral shape			
Vertebral concavity ratio L5			
** **Low	4 (33.3)	141 (37.6)	0.67 (0.18–2.55)
** **Medium	5 (41.7)	118 (31.5)	Reference
** **High	3 (25.0)	116 (30.9)	0.61 (0.14–2.61)
Spinopelvic alignment			
Pelvic incidence			
** **Low	6 (50.0)	123 (32.7)	3.10 (0.61–15.64)
** **Medium	2 (16.7)	127 (33.8)	Reference
** **High	4 (33.3)	126 (33.5)	2.02 (0.36–11.20)

Values are presented as number (%) categorical factors. This table is based on nonimputed data; missings data were 13 (3.2%) for signal intensity, 12 (2.9%) for disc height, 18 (4.4%) for Pfirrmann grade, 13 (3.2%) for nuclear shape, 18 (4.4%) for disc bulging, 20 (4.9%) for endplate irregularities, 22 (5.4%) for vertebral concavity ratio L5, and 21 (5.1%) for pelvic incidence.

LBP indicates low back pain; MRI, magnetic resonance imaging; OR, odds ratio.

## DISCUSSION

This study highlights that children with LBP at age 13 display higher odds of being male, having behavioral problems, and having pain in more than one location, as compared with children with no LBP. Meanwhile, LBP at this age is not associated with other demographic and physical characteristics, with pain at 6 years of age, or with MRI features of the spine (*i.e*. structural abnormalities, vertebral shape, and spinopelvic alignment) measured at age 9.

This study has some limitations. First, a very small proportion (2.3%) of children with data on pain exhibited LBP; this proportion is even smaller when considering the presence of LBP in those with MRI available at 9 years. This incidence estimate is lower than that reported in a previous Danish cohort study with individuals of similar age.^5^ These small absolute and relative numbers make the confidence intervals of some of the associations relatively wide (Tables 1, 2). Second, many children presented MSK pain in body areas different than the low back. Finally, we only selected the children with LBP, but we may expect to find similar associations for pain in other body parts. Nevertheless, this has not been investigated in the current study.

To the author’s knowledge, this is the first study to assess the longitudinal association between MRI features and LBP presentation in children. No association was found between MRI characteristics and LBP. However, LBP at age 13 is related to male sex, behavioral problems, and pain in other body parts. Future cohort studies with larger samples of children with LBP should attempt to replicate the findings of this study.
